# Dominance of *Bacillus* species in the wheat (*Triticum aestivum* L.) rhizosphere and their plant growth promoting potential under salt stress conditions

**DOI:** 10.7717/peerj.14621

**Published:** 2023-01-09

**Authors:** Syeda Tahseen Zahra, Mohsin Tariq, Muhammad Abdullah, Farrukh Azeem, Muhammad Arslan Ashraf

**Affiliations:** 1 Department of Bioinformatics and Biotechnology, Government College University Faisalabad, Faisalabad, Punjab, Pakistan; 2 Department of Botany, Government College University Faisalabad, Faisalabad, Punjab, Pakistan

**Keywords:** Wheat, *Bacillus*, *Bacillus tianshenii*, Biofertilizers, Salt-tolerance, Plant growth promotion

## Abstract

Wheat (*Triticum aestivum* L.) is a major source of calorific intake in its various forms and is considered one of the most important staple foods. Improved wheat productivity can contribute substantially to addressing food security in the coming decades. Soil salinity is the most serious limiting factor in crop production and fertilizer use efficiency. In this study, 11 bacteria were isolated from wheat rhizosphere and examined for salt tolerance ability. WGT1, WGT2, WGT3, WGT6, WGT8, and WGT11 were able to tolerate NaCl salinity up to 4%. Bacterial isolates were characterized *in vitro* for plant growth-promoting properties including indole-3-acetic acid (IAA) production, phosphate solubilization, nitrogen fixation, zinc solubilization, biofilm formation, and cellulase-pectinase production. Six isolates, WGT1, WGT3, WGT4, WGT6, WGT8, and WGT9 showed IAA production ability ranging from 0.7–6 µg m/L. WGT8 displayed the highest IAA production. Five isolates, WGT1, WGT2, WGT5, WGT10, and WGT11, demonstrated phosphate solubilization ranging from 1.4–12.3 µg m/L. WGT2 showed the highest phosphate solubilization. Nitrogen fixation was shown by only two isolates, WGT1 and WGT8. Zinc solubilization was shown by WGT1 and WGT11 on minimal media. All isolates showed biofilm formation ability, where WGT4 exhibited maximum potential. Cellulase production ability was noticed in WGT1, WGT2, WGT4, and WGT5, while pectinase production was observed in WGT2 and WGT3. Phylogenetic identification of potential bacteria isolates confirmed their close relationship with various species of the genus *Bacillus*. WGT1, WGT2, and WGT3 showed the highest similarity with *B. cereus*, WGT6 with *B. tianshenii*, WGT8 with *B. subtilis*, and WGT11 with *B. thuringiensis*. Biofertilizer characteristics of salt-tolerant potential rhizospheric bacteria were evaluated by inoculating wheat plants under controlled conditions and field experiments. *B. cereus* WGT1 and *B. thuringiensis* WGT11 displayed the maximum potential to increase plant growth parameters and enhance grain yield by 37% and 31%, respectively. Potential bacteria of this study can tolerate salt stress, have the ability to produce plant growth promoting substances under salt stress and contribute significantly to enhance wheat grain yield. These bacterial isolates have the potential to be used as biofertilizers for improved wheat production under salinity conditions and contribute to the sustainable agriculture.

## Introduction

Wheat (*Triticum aestivum* L.) is a staple food for several countries and approximately 35% global population feeds on wheat as a major source of calorific intake in its various forms (Kumar, Maurya & Raghuwanshi, 2021). Wheat is an annual monocotyledon crop that is cultivated in different climates throughout the world with more than 20,000 known varieties (Kavamura et al., 2021). Wheat is cultivated at the largest scale in Pakistan and its production contributes 1.7% of the country’s GDP and 9.1% in agriculture production (Mahmood et al., 2019). The annual wheat production of Pakistan is 27 million tons (Economic Survey of Pakistan 2020–2021, https://www.finance.gov.pk/survey/chapters_21/02-Agriculture.pdf). Due to climate change and unexpected rise in world population various abiotic stress like water stress, soil salinity and excessive use of chemical fertilizers have been increased that put a negative impact on plant growth which results in an approximately 50% decrease in crop yield production worldwide (Omara et al., 2022).

Soil salinity is a major limiting factor to crop growth and productivity (Allakhverdiev et al., 2000; Zhao et al., 2022). A variety of biochemical and physiological alterations in crops are induced by soil salinity *(Kamran et al., 2020; Alkharabsheh et al., 2021*). Worldwide soil salinity becomes one of the major limiting factors for food production and crop productivity. It is estimated that annually 20–50% loss of crops is due to soil salinity and drought (Ha-Tran et al., 2021). Chemical fertilizers are used to improve plant growth and crop productivity. Extensive use of chemical fertilizers has resulted in negative impacts on both human health and natural ecosystems. Biofertilizers are cost-effective and ecofriendly alternative of chemical fertilizers which contain beneficial microorganisms and can be applied to the soil or seed surfaces to promote plant growth by improving nutrient availability to plants (Bhardwaj et al., 2014; Simarmata et al., 2016; Imran, Amanullah & Al Tawaha, 2021; Kumar & Brar, 2021). Symbiotic plant growth promoting rhizobacteria (PGPR) formulations enrich soil microbiome and have ability to improve wheat growth (Dal Cortivo et al., 2020).

PGPR are usually defined as microorganisms that can grow around plant tissues. PGPR can improve plant growth directly by production of phytohormones to facilitate nutrient uptake from soil and indirectly by biocontrol mechanism (*Kohler et al., 2006)*. The direct mechanisms are associated with an increase in availability of nutrients and include biological nitrogen fixation (BNF) (Graham & Vance, 2000), phosphate solubilization (Valverde et al., 2007), siderophore production (Persmark et al., 1993), and synthesis of plant hormones such as indole acetic acid (Costacurta & Vanderleyden, 1995). Higher levels of salts in soils affects the growth and efficiency of PGPR (Roomi et al., 2002). The effectiveness of biofertilizers is adversely affected by higher levels of salinity (Kohler, Caravaca & Roldán, 2010). Better crop productivity can be achieved using salt-tolerant biofertilizers.

Some rhizospheric bacteria have the ability to tolerate salt stress and survive in osmotic and ionic environments. These salt-tolerant bacteria have ability to produce compatible solutes, osmoprotectants, and specialized transporters to adapt to salt stress and enhance plant growth. These salt-tolerant PGPR (ST-PGPR) are used as biofertilizers to improve soil fertility and enhance crop yield (Egamberdieva et al., 2019; Haroon et al., 2022). Excess of salts in soil or water is harmful to plant health. Higher concentrations of salt adversely affect the physiological process of plants including respiration, denitrification, decomposition, nitrification, and microbial activity (Schirawski & Perlin, 2018). ST-PGPR have the potential to improve plant growth in salt stress conditions and has an important place in agriculture (Niu et al., 2018). Members of the genus *Pseudomonas*, *Bacillus*, *Enterobacter*, *Agrobacterium*, *Streptomyces*, *Klebsiella*, and *Ochromobacter* are best reported for improving the productivity of diverse crops under saline conditions (Singh & Jha, 2016; Sharma et al., 2017; Sarkar et al., 2018).

Selection of salt-tolerant microbiota may play a pivotal role in producing successful biofertilizers (Egamberdieva et al., 2019). In this study, wheat salt-tolerant bacteria were isolated, phylogenetically identified, and tested *in vitro* and *in vivo* for plant growth-promoting potential under salt stress conditions.

## Materials and Methods

### Sample collection and isolation from rhizospheric zones of root

Wheat (*Triticum aestivum* L.) 8-week-old plants were sampled from cultivation site of Khewra (GPS coordinates at 32°38′52.56″ N and 73°0′30.22″ E), Salt Range, Pakistan. Under aseptic conditions, roots were washed with sterilized water to remove the loosely adhered soil. Intact roots were separated from the plants by sterilized forceps and 1 g root was placed in 9 mL of sterilized saline (0.85% NaCl) glass tube. The suspension was vigorously mixed by vortexing and diluted serially to 10^−8^ dilutions. A 100 µL aliquot of each dilute suspension was plated on LB agar plates and incubated at 28 °C for 24 h (Shahid et al., 2015; Tsegaye et al., 2019a). Bacterial colonies displaying different morphology were selected and subcultured until culture purity is confirmed. Bacterial cultures were streaked on LB agar plates and incubated for 24 h at 28 °C, and mucoid colonies were identified as gum production (Adamu-Governor et al., 2018). Bacterial cell suspension was prepared in saline and observed for size and shape of bacteria under a light microscope. Gram’s reaction was performed according to Wang, Zhu & Chen (2017).

### Salinity tolerance

Bacterial isolates were screened for salt tolerance at different levels of salts according to Verma et al. (2020), with some modifications. LB broth was prepared 20 mL in 50 mL flasks by adding different concentration of salt ranging from 0.5%, 0.1%, 1.5%, 2%, 3% and 4% (w/v) NaCl. A total of 0.5 mL bacterial culture was inoculated in each flask and incubated at 28 °C shaking at 150 rpm. Bacterial culture broth without NaCl was used as a control for comparison. Bacterial growth optical density (OD) was determined every 6 h up to 48 h at 600 nm using a spectrophotometer and compared with control optical density to examine salt tolerance (Patil et al., 2014).

### Characterization for biofertilizer attributes

#### Indole-3-acetic acid production

Bacterial isolates were tested to produce indole-3-acetic acid (IAA) by using Salkowski’s calorimetric assay. Bacterial cultures were grown in 2% (w/v) NaCl LB media, supplemented with L-tryptophan (100 µg/mL) and incubated at 28 °C for 48 h. After incubation, cultures were centrifuged for 10 min at 12,000 rpm. One mL of supernatant was vigorously mixed with 4 mL of Salkowski’s reagent (7.5 mL 0.5 M FeCl_3_.6H_2_O, 150 mL H_2_SO_4_, 250 mL H_2_O) and incubated at room temperature for 30 min (Bhattacharyya et al., 2020). Production of IAA was indicated by pink coloration, which was measured as OD at 530 nm. OD of various concentrations of IAA standard was also measured to compare and calculated sample concentration (Myo et al., 2019; Hyder et al., 2020).

#### Phosphate solubilization

Phosphate solubilization screening was performed by spotting single colonies of each bacterial isolate in the center of 2% (w/v) NaCl Pikovskaya’s agar plate containing tricalcium phosphate and incubated at 28 °C for 6 days. Plates were observed for halo zone formation around colonies (Linu et al., 2019; Oo et al., 2020). Phosphate solubilization was quantified by Phospho-molybdate blue color method. Bacterial isolates were grown in 2% (w/v) NaCl Pikovskaya’s broth medium and incubated at 28 ± 2 °C for 6 days. After incubation, 1 mL of supernatant was mixed with 0.2 mL phospho-molybdate reagent (containing H_2_O 46.5 mL, H_2_SO_4_(5N) 3.5 mL, (NH_4_)_6_Mo_7_O_24_ 300 g, C_6_H_8_O_6_ 264 mg, K_2_Sb_2_C_8_H_4_O_12_.3H_2_O 6.9 mg) and incubated for 10 min. Absorbance was recorded at 880 nm using a spectrophotometer. The amount of P solubilized by the bacterial isolates was determined by comparing data with standard curve generated from the standard solution of P (Behera et al., 2017).

#### Nitrogen fixation

Nitrogenase activity was measured by acetylene reduction assay on a gas chromatograph using a Porapak Q column and an H_2_-flame ionization detector. Nitrogen fixation ability of bacterial strains was assessed by inoculating individual colonies in 5 mL semi-solid 2% (w/v) NaCl nitrogen-free media (NFM) in 15 mL vials and incubating at 28 °C for 48 h. Vials were injected with acetylene (10% v/v) and incubated at room temperature for 16 h and 100 µL gas samples were analyzed on a gas chromatograph (Gómez-Godínez et al., 2019; Wang et al., 2020).

#### Zinc mobilization

*In vitro* zinc solubilization ability of bacterial isolates was measured according to Ramesh et al. (2014), with some modifications. Bacterial isolates were grown on 2% (w/v) NaCl-Tris-minimal agar (TMA) medium supplemented with D-glucose and different insoluble zinc compounds. The Tris-minimal medium was separately amended with 15.23 mM zinc oxide (ZnO) and 5.2 mM zinc carbonate (ZnCO_3_) to a concentration equivalent to 0.1% zinc. Freshly grown bacterial cultures were spot inoculated on supplemented TMA plates and incubated at 28 °C for 7 days in the dark. Plates were observed for halo zone formation around colonies. The diameter of the halo zone around the colonies and colonies’ diameters were measured. Zinc solubilization efficiency (SE) was determined as SE = (diameter of solubilization halo/diameter of the colony) × 100 (Rezaeiniko, Enayatizamir & Norouzi Masir, 2022).

#### Biofilm formation assay

Biofilm formation was performed by using a microtiter plate according to Kasim et al. (2016), with some modifications. Bacterial cultures were grown in 2% (w/v) NaCl LB broth media up to an optical density of 2.0 measured by spectrophotometer at 600 nm wavelength. Bacterial cultures were centrifuged at 8,000 rpm for 2 min, supernatant was discarded, and the pellet was rinsed with sterilized water. The cells were resuspended in the same media and diluted to an OD_600_ = 0.2. An aliquot (150 µL) of bacterial cell suspension was added to each well of 96-well polyvinyl chloride (PVC) plate. Plates were incubated at 28 °C for 24 h after covering with a plastic lid. The medium was removed after the incubation period and the wells were rinsed with sterile water. Wells were stained with 150 µL of 0.001% crystal violet. After removing the dye, the wells were rinsed with sterile water. Then 150 µL of 95% ethanol was added to solubilize crystal violet stain and the amount of dye was measured using a plate reader at the wavelength of 570 nm (Sampedro et al., 2020).

#### Cellulase production assay

Bacterial isolates were spot inoculated onto 2% (w/v) NaCl carboxymethyl cellulose (10 g/L) agar plates (Islam & Roy, 2018) and incubated at 28 °C for 3 days. Plates were stained with 0.2% Congo red dye and incubated at 30 °C for 15 min and washed with 1 M NaCl. The production of halo zone around the colonies indicated cellulolytic activity of bacterial isolates (Suárez-Moreno et al., 2019).

#### Pectinase production assay

Bacterial isolates were spot inoculated onto 2% (w/v) NaCl pectin (10 g/L) agar plates (Devi et al., 2022) and incubated at 28 °C for 7 days. After incubation, the plates were stained with 1% iodine solution for 15 min, then washed with water. Pectinase production was indicated by the formation of halo zone around the colonies (Tsegaye et al., 2019b).

#### Phylogenetic identification of bacterial isolates

Bacterial isolates were phylogenetically identified according to La Pierre et al. (2017), with some modifications. The 16S rRNA was amplified by using universal primer primers fD1 (5′-AGAGTTTGATCCTGGCTCAG-3′) and rD1 (5′–AAGGAGGTGATCCAGCC-3′) (Weisburg et al., 1991). A total of 25 µL PCR reaction mixture was prepared by using the recipe (10× Taq polymerase buffer 2.5 μL, primers (10 pmoles/100 μL) 2 μL each, 2 mM dNTPs 2.5 μL, 25 mM MgCl_2_ 2 μL, H_2_O 11.7 μL, Taq polymerase enzyme (concentration of 5U/μL) 0.3 μL, template DNA (20 ng/μL) 2 μL). A reaction mixture of 25 µL was prepared for 16S rRNA gene amplification for each isolate and denatured in a thermal cycler for 5 min, followed by 30 cycles of denaturation at 94 °C for 60 s, primer annealing at 55 °C for 50 s, primer extension at 72 °C for 1 min 40 s, and final extension at 72 °C for 5 min. After amplification, the amplified product was examined on 1% agarose gel and visualized under UV in a gel documentation system. The amplicons were purified using PCR purification kit (GeneJET PCR Purification Kit catalog number K0701; Thermo Fisher Scientific, Waltham, MA, USA) and Sanger sequenced using commercial service of Macrogen Inc, (Seoul, South Korea). Sequence contigs were made by assembling the sequences. Using NCBI BLAST tool, the gene sequences were compared with database sequences (Altschul et al., 1990). Closely related authentic sequences were retrieved from public databases and pairwise sequence comparisons were done using Sequence Demarcation Tool (SDT) v.1.2 (Zhang et al., 2000; Muhire, Varsani & Martin, 2014). Phylogenetic analysis was conducted by constructing a phylogenetic tree using the maximum likelihood algorithm as implemented by MEGA 11 with 1,000 bootstrap values (Kumar, Stecher & Tamura, 2016; Noori et al., 2021).

### Controlled condition experiment

A pot experiment was conducted on wheat (*Triticum aestivum* L.) cultivar V-17086 with eight treatments and four replicates using a completely randomized design (CRD). Treatments include six biofertilizer potential bacteria (WGT1, WGT2, WGT3, WGT6, WGT8, and WGT11), bacterial consortia (mixture of all six biofertilizer potential bacteria), and water as control. Selected salt-tolerant bacterial isolates of wheat were grown in LB broth, centrifuged at 6,000 rpm and pellet was dissolved in sterile water to dilute bacteria to OD 0.5 (Mishra et al., 2009). Seeds were surface sterilized with 75% ethanol for 1 min and 3% solution of calcium hypochlorite for 5 min and washed thoroughly with sterile water. For germination, surface sterilized seeds were placed on moist filter paper in Petri dishes and kept in a dark room at 25 °C ± 2 for 2 days. Germinated seeds of uniform size (2 cm) were transplanted in pots containing 2% NaCl sterile soil and applied with 100 μL of each treatment. Daily 5 mL of quarter-strength Hoagland’s solution was applied alternating with sterilized water to each plant. Pots were incubated in a growth chamber at 30 ± 2 °C during the day and 20 ± 2 °C at night. Plants were harvested after 6 weeks, plants were observed for the appearance of any pathogenic effect, including the development of sclerotia, lesion and chlorosis and agronomical parameters were examined like dry weight per plant, fresh weight per plant, root length, and shoot length (Tounsi-Hammami et al., 2022). The data was analyzed statistically using CoStat software (Cardinali & Nason, 2013).

### Field experiment

Field experiment was carried out on wheat (*Triticum aestivum* L.) cultivar V-17086 by using randomized complete block design (RCBD) with eight treatments and three replicates at university field area (GPS coordinates at 31°16′40.2″ N and 72°19′20.6″ E), Faisalabad, Pakistan. Field was previously under cultivation of wheat and rice and soil has electrical conductivity (EC_e_) of 2.1 ± 0.15 dS/m. Treatments included six biofertilizer potential bacteria (WGT1, WGT2, WGT3, WGT6, WGT8, and WGT11), bacterial consortia (mixture of all six biofertilizer potential bacteria), and water as control. Each replicate comprised of a row containing 30 plants. Inoculums of bacterial strains were prepared as mentioned in pot experiment and diluted to OD 0.5. After 2 weeks of germination, 100 μL of bacterial inoculum was applied to the stem base and roots of each plant. The second inoculation was applied after 6 weeks of germination. Field was watered with canal water having EC_e_ 1.17 ± 0.19 as and when required. After 120 days, mature plants were harvested and agronomical parameters including plant height, spike length, numbers of spikelets, number of seeds per plant and grain yield were recorded (Kumar, Maurya & Raghuwanshi, 2021). Field data were analyzed statistically using CoStat software (Cardinali & Nason, 2013).

## Results

### Isolation of bacteria

Eleven bacterial morphotypes were selected based on colony size, shape, color, edges, surface and gum production. All bacterial cells were rod-shaped, except WGT10, which showed circular cell shape. All bacteria were Gram positive, except WGT7 and WGT10. Colony and cell characteristics of selected wheat rhizospheric bacteria are mentioned in Table 1.

**Table 1:** Colony & cell morphology, gum production and Gram staining of wheat rhizosphere-associated bacteria.

Isolates	Colony morphology	Cell morphology	Gum production	Gram staining
WGT1	Medium, Circular, Light Brown, Smooth, Flat	Rod	+	+
WGT2	Very small, Circular, Light Brown, Wavy, Flat	Rod	+	+
WGT3	Small, Circular, Brown, Smooth, Flat	Small rod	–	+
WGT4	Medium, Circular, Milky White, Smooth, Flat	Rod	+	+
WGT5	Small, Circular, Transparent white, Smooth, Flat	Small rod	+	+
WGT6	Medium, Irregular, Light brown, Wavy, Rough	Rod	–	+
WGT7	Small, Irregular, Transparent white, Wavy, Flat	Small rod	–	–
WGT8	Medium, Circular, Milky white, Wavy, Flat	Rod	+	+
WGT9	Small, Circular, Light brown, Smooth, Normal	Rod	–	+
WGT10	Medium, Irregular, Light yellow, Wavy, Rough	Circular	–	–
WGT11	Large, Irregular, White, Wavy, Flat	Rod	+	+

### Salinity response of wheat isolates

**Figure 1:**
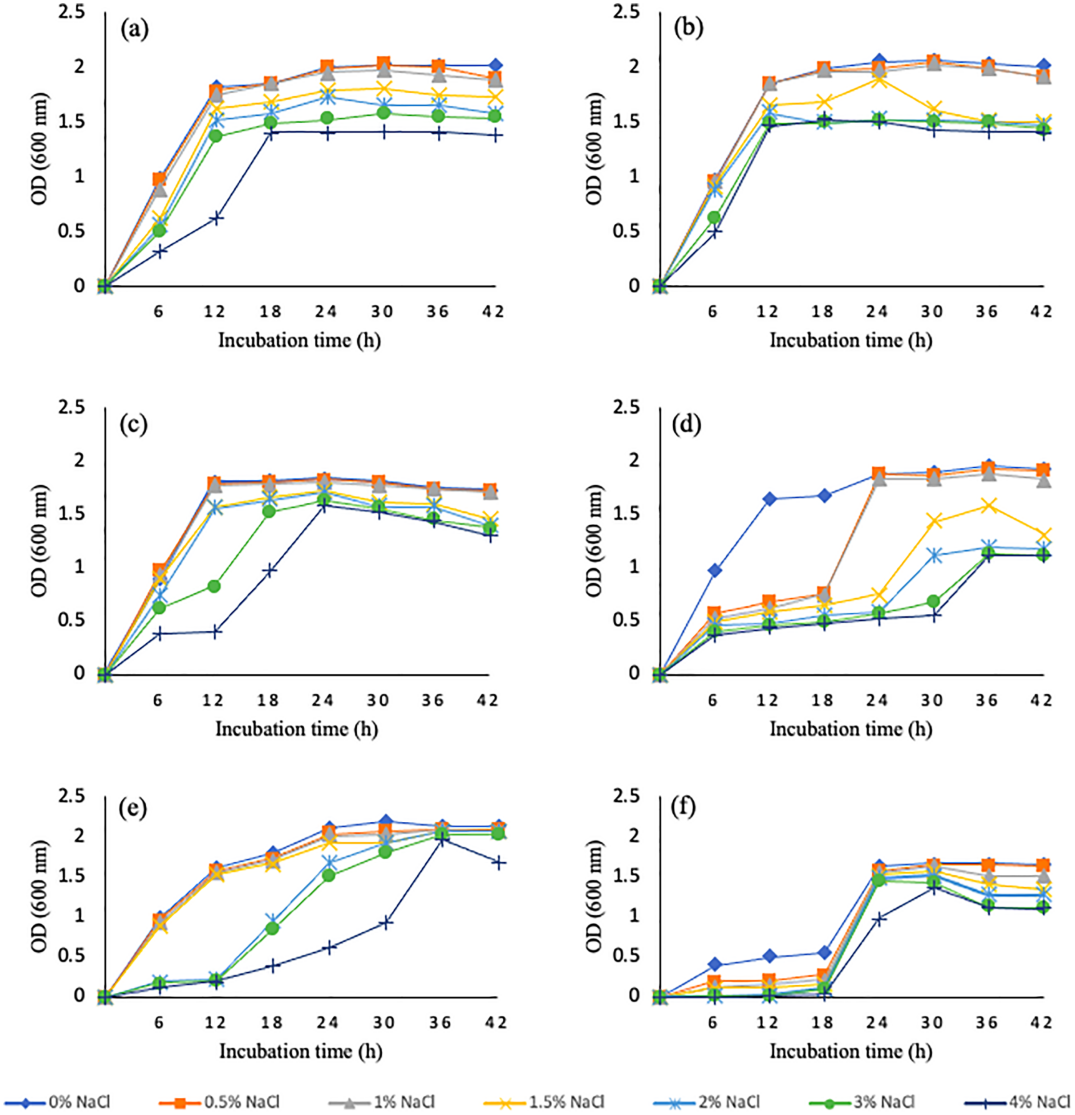
Graphical representation of wheat rhizosphere associated bacteria to tolerate varying salt concentrations. WGT1 (A), WGT2 (B), WGT3 (C), WGT6 (D), WGT8 (E) and WGT11 (F) showed considerable growth upto 4% NaCl.

Salinity tolerance ability of wheat rhizosphere bacterial isolates was tested at different levels of NaCl concentrations ranging from 0.5–4% NaCl. Six bacterial isolates, WGT1, WGT2, WGT3, WGT8, WGT6, and WGT11 were able to tolerate salinity levels and showed significant growth in NaCl stress (Fig. 1). Rest of the five isolates, WGT4, WGT5, WGT7, WGT9, and WGT10, significantly inhibited by different salinity levels.

### Screening of biofertilizer characteristics

The quantity of the IAA production was estimated based on the intensity of pink color production using spectrophotometer. Out of 11 isolates of wheat, six isolates WGT1, WGT3, WGT4, WGT6, WGT8, and WGT9 showed IAA production ranging from 0.7–6 µg m/L (Table 2). WGT8 showed the highest IAA production, whereas WGT4 exhibited the lowest potential.

**Table 2:** *In vitro* testing of wheat rhizosphere associated bacteria for plant growth promoting attributes.

Isolates	Phosphate solubilization (µg m/L)	IAA production (µg m/L)	Nitrogen fixation (n mole C_2_H_2_ reduced h^−1^mg^−1^ protein)	Zinc (ZnO) solubilization efficiency (%)	Zinc (ZnCO_3_) solubilization efficiency (%)	Biofilm formation activity (OD 570 nm)	Cellulase activity	Pectinase activity
WGT1	8.9 ± 0.26	4.9 ± 0.14	66.8 ± 3.1	250	200	0.212 ± 0.016	+	–
WGT2	12.3 ± 0.82	–	–	–	–	0.395 ± 0.013	+	+
WGT3	–	3.6 ± 0.22	–	–	–	0.735 ± 0.045	–	++
WGT4	–	0.7 ± 0.1	–	–	–	1.77 ± 0.029	+++	–
WGT5	1.4 ± 0.14	–	–	–	–	0.436 ± 0.024	++	–
WGT6	–	3.6 ± 0.29	–	–	–	0.171 ± 0.013	–	–
WGT7	–	–	–	–	–	0.245 ± 0.018	–	–
WGT8	–	6 ± 0.25	56.2 ± 2.5	–	–	0.709 ± 0.007	–	–
WGT9	–	2.7 ± 0.25	–	–	–	0.513 ± 0.027	–	–
WGT10	1.7 ± 0.1	–	–	–	–	0.435 ± 0.015	–	–
WGT11	3.8 ± 0.22	–	–	200	176	0.279 ± 0.022	–	–

**Note:**

(–) Presents no production, (+) presents weak production, (++) presents moderate production, (+++) presents high production.

The amount of phosphate solubilization was quantified by Phospho-molybdate blue color method presented in Table 2. Out of 11 bacterial isolates, only five isolates WGT1, WGT2, WGT5, WGT10, and WGT11 indicated phosphate solubilization ranging from 1.4–12.3 µg m/L. WGT2 showed the highest phosphate solubilization of 12.3 µg m/L, whereas WGT5 showed the lowest potential.

Nitrogenase activity in acetylene reduction assay of wheat rhizobacteria was shown by only two isolates WGT1 and WGT8 among all isolates of wheat. WGT1 has the highest ability of nitrogenase activity by reducing 66.8 n mole C_2_H_2_/h/mg protein (Table 2).

Out of 11 isolates of wheat, only two isolates of wheat WGT1 and WGT11 showed zinc solubilization on tris-minimal media supplemented with zinc oxide and zinc carbonate. WGT1 showed higher solubilization efficiency of 250% in zinc oxide and 200% in zinc carbonate (Table 2).

All isolates of wheat showed varying biofilm formation ability ranging OD 0.17–1.77. WGT4 exhibited the highest biofilm formation ability and WGT6 showed the least ability (Table 2).

Cellulase and pectinase activity was checked by measuring the halo zone produced by the bacterial isolate on the plate. Out of 11 isolates of wheat only four isolates WGT1, WGT2, WGT4, and WGT5 showed cellulase activity. WGT4 showed the highest cellulase production among all isolates (Table 2).

Only two isolates WGT2 and WGT3 showed pectinase activity. WGT3 showed better pectinase production potential (Table 2).

### Phylogenetic identification of potential bacteria

**Figure 2:**
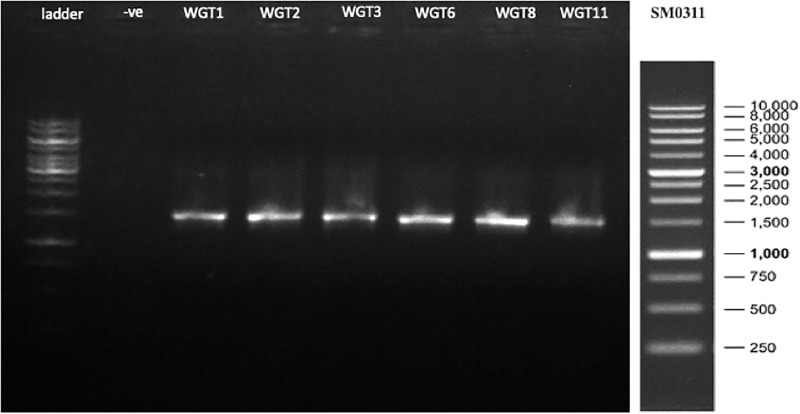
16S rRNA gene amplification of potential isolates. A total of 1,500 bp amplicons were produced by polymerase chain reaction.

**Figure 3:**
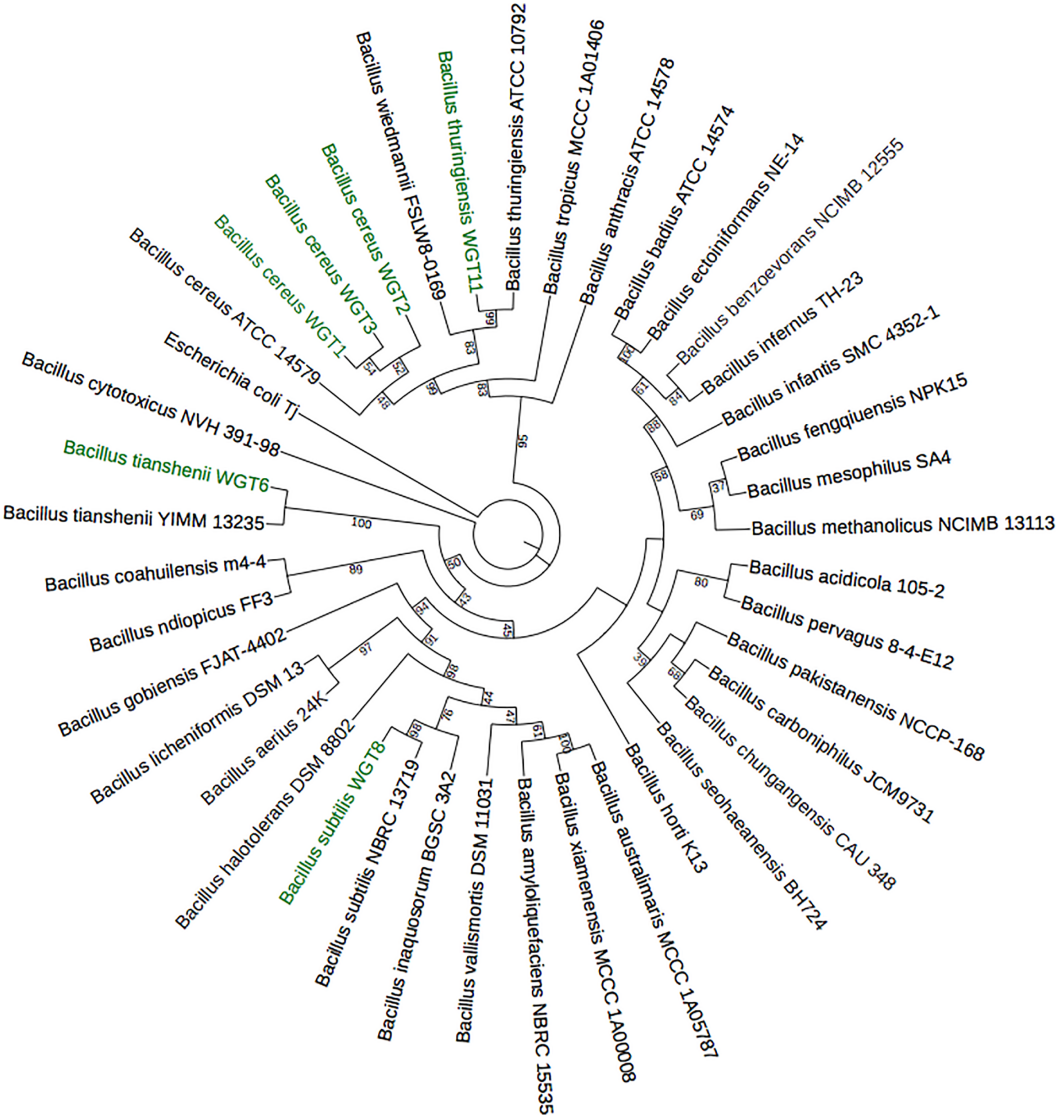
Phylogenetic tree of wheat rhizosphere associated bacteria with authenticated sequences of *Bacillus*. WGT1, WGT2 and WGT3 positioned in the neighborhood of *Bacillus cereus*, WGT8 in *Bacillus subtilis*, WGT6 in *Bacillus tianshenii* and WGT11 in *Bacillus thuringiensis*.

**Figure 4:**
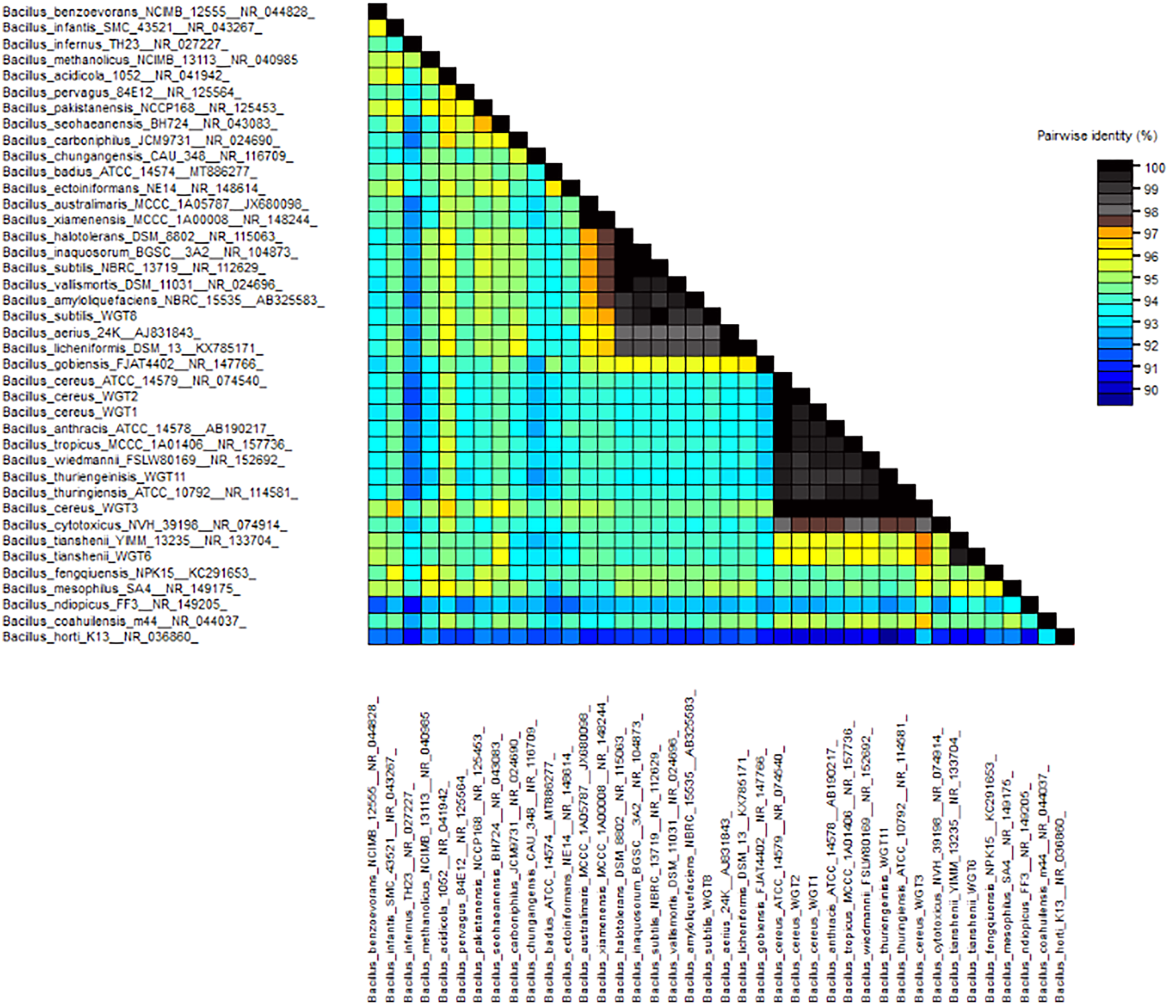
Pairwise identity chart of wheat rhizosphere associated bacteria with authenticated sequences of *Bacillus*. WGT1, WGT2 and WGT3 showed 99.9%, 99.9% and 100%, respectively, identity with *Bacillus cereus*. WGT6, WGT8 and WGT11 showed 100% identify with *Bacillus tianshenii*, *Bacillus subtilis* and *Bacillus thuringiensis*, respectively.

A total of 1,500 bp DNA band was produced from the primers (Fig. 2). The purified PCR products were sequenced from Macrogen and the final contigs were compared at NCBI BLAST tool with available sequence data to check maximum similarity for phylogenetic identification. Phylogenetic analysis of potential isolates revealed a close relationship with various species of *Bacillus*. WGT1, WGT2, and WGT3 showed the highest similarity with *Bacillus cereus*, WGT6 with *Bacillus tianshenii*, WGT8 with *Bacillus subtilis*, and WGT11 with *Bacillus thuringiensis* shown in Table 3. A phylogenetic tree of 16S rRNA gene sequence of potential bacteria was constructed with forty different authenticated species of genus *Bacillus* and one out-group *Escherichia coli* B2GNS10-1. The selected *Bacillus* sp. fall into three clades which belongs to common ancestors. WGT1, WGT2, WGT3, and WGT11 were positioned in clade-1, WGT6, and WGT8 in clade-3. WGT1, WGT2, and WGT3 were found in the neighborhood of *Bacillus cereus*, WGT8 in *Bacillus subtilis*, WGT6 in *Bacillus tianshenii* and WGT11 was the closed neighbor of *Bacillus thuringiensis* (Fig. 3). A color-coded pairwise identity matrix was also created, in which each colored cell represents the percentage identity of two sequences. The similarity between the sequences ranges from 90–100% (Fig. 4).

**Table 3:** Phylogenetic identification of wheat rhizosphere associated bacteria.

Bacterial strain	Taxonomic identification	Percent identity (%)	GenBank accession number
WGT1	*Bacillus cereus*	99.93	OP388443
WGT2	*Bacillus cereus*	99.93	OP388444
WGT3	*Bacillus cereus*	100	OP388445
WGT6	*Bacillus tianshenii*	100	OP388446
WGT8	*Bacillus subtilis*	100	OP388447
WGT11	*Bacillus thuriengiensis*	100	OP388448

### Controlled condition experiment

There were no sclerotia, lesion and chlorosis spots observed on treated plants. WGT1, WGT6 and WGT11 showed a significant increase in root length compared to control. WGT1, WGT6, WGT8 and WGT11 have a significant potential to improve shoot length. In case of plant fresh weight all potential isolates except WGT3 and consortia performed best. WGT1 and WGT11 exhibited significant increase in plant dry weight compared to control shown in Table 4. WGT1 showed the highest potential to increase root length by 33%, shoot length by 53%, plant fresh weight by 52%, and plant dry weight by 48% compared with the control. Consortia and WGT3 did not show any positive effect in improving plant growth parameters.

**Table 4:** Effect of inoculation of wheat bacterial isolates on plant growth under controlled condition experiment.

Treatment	Root length (cm)	Shoot length (cm)	Fresh weight (mg)	Dry weight (mg)
Control	10.62 ± 0.75^c^	27.75 ± 0.97^e^	690 ± 60^d^	327 ± 20^b^
WGT1	14.12 ± 0.5^a^	42.67 ± 1.3^a^	1050 ± 20^a^	490 ± 18^a^
WGT2	11.25 ± 0.3^bc^	30.5 ± 0.5^de^	970 ± 17^ab^	430 ± 19^a^
WGT3	9.75 ± 0.5^c^	24.5 ± 1.9^f^	750 ± 20^cd^	280 ± 14^bc^
WGT6	12.75 ± 0.6^ab^	33.9 ± 1.2^c^	950 ± 16^ab^	350 ± 19^bc^
WGT8	10.75 ± 0.3^c^	31.1 ± 0.7^cd^	860 ± 11^bc^	240 ± 11^c^
WGT11	13.25 ± 0.6^a^	37.5 ± 0.4^b^	990 ± 55a^a^	450 ± 11^a^
Consortia	8 ± 0.4^d^	22.4 ± 0.4^f^	430 ± 9^e^	150 ± 9^d^
LSD (0.05)	1.55	3.16	110	87
ANOVA significance	***	***	***	***

**Note:**

LSD, least significant difference. Each value represents mean (*n* = 4) ± standard error. Values followed by the different letters in same column indicate significant difference and followed by same letters are not significantly different. (***) indicates highly significant.

### Field trials of wheat

The effect of potential rhizospheric bacteria WGT1, WGT2, WGT3, WGT6, WGT8, WGT11, and consortia were examined in a field experiment. Mature plants were harvested, and agronomical parameters were calculated and analyzed statistically summarized in Table 5. There is non-significant effect of different treatments on plant height compared to the control. WGT6, WGT8, WGT11 and consortia improved spike length significantly compared to control. WGT1 and WGT11 showed a significant increase in the number of spikelets per spike compared to control. All potential isolates exhibited significant increase in the number of seeds per plant compared to control. WGT1, WGT6 and WGT11 showed the maximum potential to increase grain yield compared to control. WGT1 exhibited highest potential to enhance grain yield by (37%).

**Table 5:** Effect of inoculation of wheat bacterial isolates on plant growth in field trials.

Treatment	Plant height (cm)	Spike length (cm)	No. of spikelet per spike	No. of seeds per plant	Grain yield per plant (g)
Control	91.1 ± 4.8^ab^	8.4 ± 0.47^cd^	15.2 ± 0.77^cd^	79.8 ± 5.06^c^	3.2 ± 0.22^d^
WGT1	94.3 ± 3.4^ab^	9.3 ± 0.34^abc^	18.2 ± 0.52^a^	120 ± 5.44^a^	4.4 ± 0.33^a^
WGT2	94.3 ± 2.4^ab^	8.4 ± 0.25^d^	14.2 ± 0.66^d^	98.2 ± 2.65^b^	3.3 ± 0.12^cd^
WGT3	93.5 ± 2.5^ab^	8.7 ± 0.39^bcd^	16.2 ± 0.52^bc^	120 ± 5.44^a^	3.9 ± 0.32^abcd^
WGT6	88.8 ± 2.5^b^	9.5 ± 0.31^ab^	16.3 ± 0.54^bc^	110 ± 6.8^ab^	4.0 ± 0.28^abc^
WGT8	95.3 ± 1.7^ab^	9.4 ± 0.43^ab^	15.8 ± 0.77^bc^	120 ± 7.78^a^	3.6 ± 0.33^bcd^
WGT11	97 ± 1.9^a^	9.7 ± 0.11^a^	17.2 ± 0.66^ab^	118.7 ± 4.27^a^	4.2 ± 0.31^ab^
Consortia	94.8 ± 1.3^ab^	9.6 ± 0.23^a^	15.5 ± 0.47^cd^	106.2 ± 4.98^ab^	3.5 ± 0.2^bcd^
LSD (0.05)	7.21	0.852	1.62	14.3	0.72
ANOVA significance	ns	**	***	***	*

**Note:**

LSD, least significant difference. Each value represents mean (*n* = 6) ± standard error. Values followed by the different letters in same column indicate significant difference and followed by same letters are not significantly different. (ns) indicates no significant, (*) indicates significant, (**) indicates moderately significant, (***) indicates highly significant.

## Discussion

Extensive use of chemical fertilizers has resulted in negative impacts on both human health and natural ecosystems. Biofertilizer is a cost-effective and environment-friendly way to increase plant growth and produce healthy food in a sustainable manner (Dal Cortivo et al., 2020). Efficiency of biofertilizers reduces due to saline environment and desired crop productivity is not achieved (Roomi et al., 2002). Salinity alters the physiochemical processes of the plant and reduces plant growth (Saddiq et al., 2021). Several salt-tolerant bacteria have potential to improve soil fertility and crop productivity through several mechanisms including hormones production, growth factors and vitamins production under salt stress (Arora et al., 2020; Mahapatra, Yadav & Ramakrishna, 2022). In this study, salt-tolerant rhizospheric bacteria were isolated from the wheat (*Triticum aestivum* L.) and tested *in vitro* and *in vivo* for plant growth promotion. Tank & Saraf (2010) reported that rhizospheric bacteria have the potential to improve tomato growth under salt stress conditions. Akram et al. (2016) also reported that some bacterial isolates from maize tolerate higher salinity levels and most of the bacterial growth was inhibited at a higher level of NaCl. Similarly, Shabaan et al. (2022) also reported the existence of salt-tolerant PGPR conferring salt tolerance in maize plants.

Biofertilizer bacteria have ability to produce several plant growth-promoting substances. Indole-3-acetic acid (IAA) has a role to reduce abiotic stress and improve root hair network (Sabki, 2021). In this study, IAA production was prominent in WGT1, WGT3, WGT4, WGT6, WGT8, and WGT9. Previously Mohite (2013) isolated rhizospheric bacteria root surface of wheat and showed that most of the rhizospheric bacteria have ability to produce IAA. Similarly, Chandra, Askari & Kumari (2018) also reported that most rhizospheric bacteria have ability to produce IAA. Our results are in agreement with Babar et al. (2021) who also reported that root-associated halo tolerant bacteria produce IAA under salt stress. Phosphate is a macromolecule, which is required for many cellular processes including photosynthesis and cell division (Khan et al., 2009). In this study, WGT1, WGT2, WGT5, WGT10, and WGT11 were the most efficient for phosphate solubilizers under saline conditions. Previously, Joshi, Kumar & Brahmachari (2021) isolated bacteria and showed that most of the bacteria have ability to solubilize phosphate under saline conditions. Gontia-Mishra et al. (2017) also reported that rhizospheric bacteria from wheat and revealed that most of the rhizospheric bacteria have ability to solubilize phosphate. Similarly, Ahmad et al. (2021) also reported that *Bacillus* species isolated from cotton rhizosphere have potential to solubilize inorganic rock phosphate and improve plant growth. Nitrogen fixation was quantified by acetylene reduction assay and only two isolates WGT1 and WGT8 have the ability to fix nitrogen. Wang et al. (2020) reported that PGPR have nitrogen fixation ability for better growth of wheat. Similarly, Peng et al. (2021) also reported that rhizospheric bacteria have the potential for nitrogen fixation under salt stress.

Two isolates WGT1 and WGT11 showed zinc solubilization on tris minimal media supplemented with zinc oxide and zinc carbonate. Ramesh et al. (2014) isolated zinc solubilizing bacteria from wheat rhizosphere and represent zinc solubilization efficiency. In this study, rhizobacteria of wheat have the ability to zinc solubilization under salt stress having solubilization efficiency of 250% in zinc oxide and 200% in zinc carbonate. Similarly, Mehmood et al. (2021) also demonstrated that rhizobacteria have ability to solubilize zinc under saline conditions. Dhaked et al. (2017) also screened zinc solubilizing bacteria from different rhizosphere having solubilization efficiency ranges from 333% to 150%.

Biofilm formation is a very important characteristic of bacteria to survive under stress conditions (Ansari & Ahmad, 2018). In this study, all bacteria showed biofilm formation ability but WGT4 showed the highest biofilm formation ability. Previously, Ansari, Ahmad & Pichtel (2019) isolated *Bacillus pumilus* from wheat rhizosphere and reported the biofilm formation ability and other plant growth-promoting characteristics under salt stress. Similarly, Yasmeen et al. (2020) also demonstrated that halotolerant bacteria have ability of biofilm formation and enhance growth of sunflower plants under salt stress.

Cellulase and pectinase belong to the hydrolytic enzyme family. These enzymes are involved in the decomposition of organic remains and biocontrol of fungal pathogens (Reetha et al., 2014). In this study, WGT2 and WGT3 showed pectinase ability and WGT2, WGT3, WGT4, and WGT5 showed cellulase production. Sebihi et al. (2016) reported that *Pseudomonas fluorescens* from the wheat rhizosphere exhibited the ability to produce cellulase and pectinase, which contribute to degrading fungal cell walls.

The potential bacteria were selected on the bases of salt tolerance and *in vitro* plant growth-promoting properties and subjected to taxonomic identification. 16S rRNA gene sequence analysis revealed that WGT1, WGT2, and WGT3 have the highest similarity with *Bacillus cereus*, WGT6 with *Bacillus tianshenii*, WGT8 with *Bacillus subtilis* and WGT11 with *Bacillus thuringiensis*. This is the first report that highlights the occurrence of *Bacillus tianshenii* in wheat rhizosphere. This study also reports the dominant existence of *Bacillus* species in the wheat rhizosphere at the Salt Range, Pakistan. These bacteria can be used as biofertilizers to promote plant growth and mitigate salt stress. Previously, Ferreira et al. (2021) reported the occurrence of *Bacillus tianshenii* in the roots of *Salicornia ramosissima* and explained its plant growth promoting attributes. *Bacillus cereus* and *Bacillus subtilis* were also reported to occupy wheat rhizosphere niches. Ashraf, Bano & Ali (2019) isolated *Bacillus cereus* from wheat rhizosphere and reported its biofertilizer effect on maize. Recently, Adeleke, Ayangbenro & Babalola (2021) also reported that *Bacillus cereus* associated with sunflower have potential to produce PGP substances and improve plant growth. Xu et al. (2022) isolated *Bacillus subtilis* from the soil of wheat rhizosphere and showed the antagonism effect against *Fusarium* crown rot fungal disease of wheat and increase the fresh and dry weight of the wheat. Cherif-Silini et al. (2016) also isolated *Bacillus subtilis* and *Bacillus thuringiensis* from the wheat rhizosphere exhibiting plant growth-promoting and biocontrol properties.

In control conditions and field trials, selected bacteria increased plant growth under saline conditions. *Bacillus cereus* WGT1 and *Bacillus thuringiensis* WGT11 showed the highest plant growth-promoting potential by increasing 37% and 31% grain yield, respectively. Delfim & Dijoo (2021) also demonstrated that *Bacillus thuringiensis* have potential to produce diverse PGP compounds for crop production and can be used as biofertilizers. Previously, Huang et al. (2022) also reported that salt-tolerant PGPR have the potential to increase plant fresh weight by 36.31% and plant length by 71.21%. Ibarra-Villarreal et al. (2021) showed that many *Bacillus* species enhance wheat growth under salt stress and reduce the adverse effects of salinity. Majeed et al. (2015) isolated *Bacillus* sp. from the rhizosphere of wheat and demonstrated their potential to enhance wheat growth. Our results are in agreement with Zhou et al. (2022), which showed that *Bacillus cereus* increases cucumber plant growth and enhances photosynthetic ability under salt stress. Similarly, Ali et al. (2022) reported that Halo-tolerant *Bacillus thuringiensis* have the potential to enhance plant growth under saline conditions. Sheirdil et al. (2019) also reported that wheat rhizosphere-associated bacteria showed an increase in root, shoot length, and yield of the plant under control conditions and field experiments. Inoculation of PGPR enhances grain yield and straw yield of wheat (Kumar, Maurya & Raghuwanshi, 2014). Our findings strengthen that *Bacillus* species dominantly exist in the wheat rhizosphere and promote plant growth under salt stress conditions. As WGT1 and WGT11 bacterial isolates exhibited the highest potential to produce plant growth promoting substance’s ability under *in vitro* assays that is why these bacteria performed considerably better in pot and field experiments, which is largely supported by literature (Baliyan et al., 2018; Sood et al., 2019; Panhwar et al., 2020; Iqbal et al., 2020; Khudhur, 2020; Dasila et al., 2022; Khan et al., 2022). Most potential bacterial isolates, *Bacillus cereus* WGT1, and *Bacillus thuringiensis* WGT11 could be used as biofertilizers to produce safe and healthy food in sustainable manner.

## Conclusions

Wheat from the salt-affected areas carry a vast array of salt-tolerant bacteria. These salt-tolerant bacteria persist their ability to produce plant growth-promoting substance in the salt-tainted environment. This study revealed the richness of *Bacillus* sp. in the wheat rhizosphere growing under salt affected soil, in which *B. cereus*, *B. tianshenii*, *B. subtilis*, and *Bacillus thuringiensis* were found the main occupants showing plant growth-promoting properties. This is the first report on the occurrence of *Bacillus tianshenii* in the wheat rhizosphere. *In vitro* and *in vivo* evaluation demonstrated magnanimous plant growth-promoting potential of *Bacillus cereus* WGT1 and *Bacillus thuringiensis* WGT11 by improving grain yield more than 30%. These salt-tolerant bacteria have the potential to be used as biofertilizers for healthy and salt-affect soil to improve crop productivity and feed the globe.

## Supplemental Information

10.7717/peerj.14621/supp-1 Supplemental Information 1 OD reading raw data.

10.7717/peerj.14621/supp-2 Supplemental Information 2 Gel pic raw data.

10.7717/peerj.14621/supp-3 Supplemental Information 3 PGP characteristics raw data.

10.7717/peerj.14621/supp-4 Supplemental Information 4 Controlled condition experiment of wheat raw data.

10.7717/peerj.14621/supp-5 Supplemental Information 5 Field trials raw data.

## Additional Information and Declarations

### DNA Deposition

The following information was supplied regarding the deposition of DNA sequences: The sequences are available at GenBank: OP388443, OP388444, OP388445, OP388446, OP388447, OP388448.

### Data Availability

The following information was supplied regarding data availability: The raw data is available in the Supplemental Files.
