# Supplementary figures and images for: Dominance of Bacillus species in the wheat (Triticum aestivum L.) rhizosphere and their plant growth promoting potential under salt stress conditions
Source: PeerJ. 2023 Jan 9;11:e14621. doi: 10.7717/peerj.14621 (PMC9835707; doi:10.7717/peerj.14621)

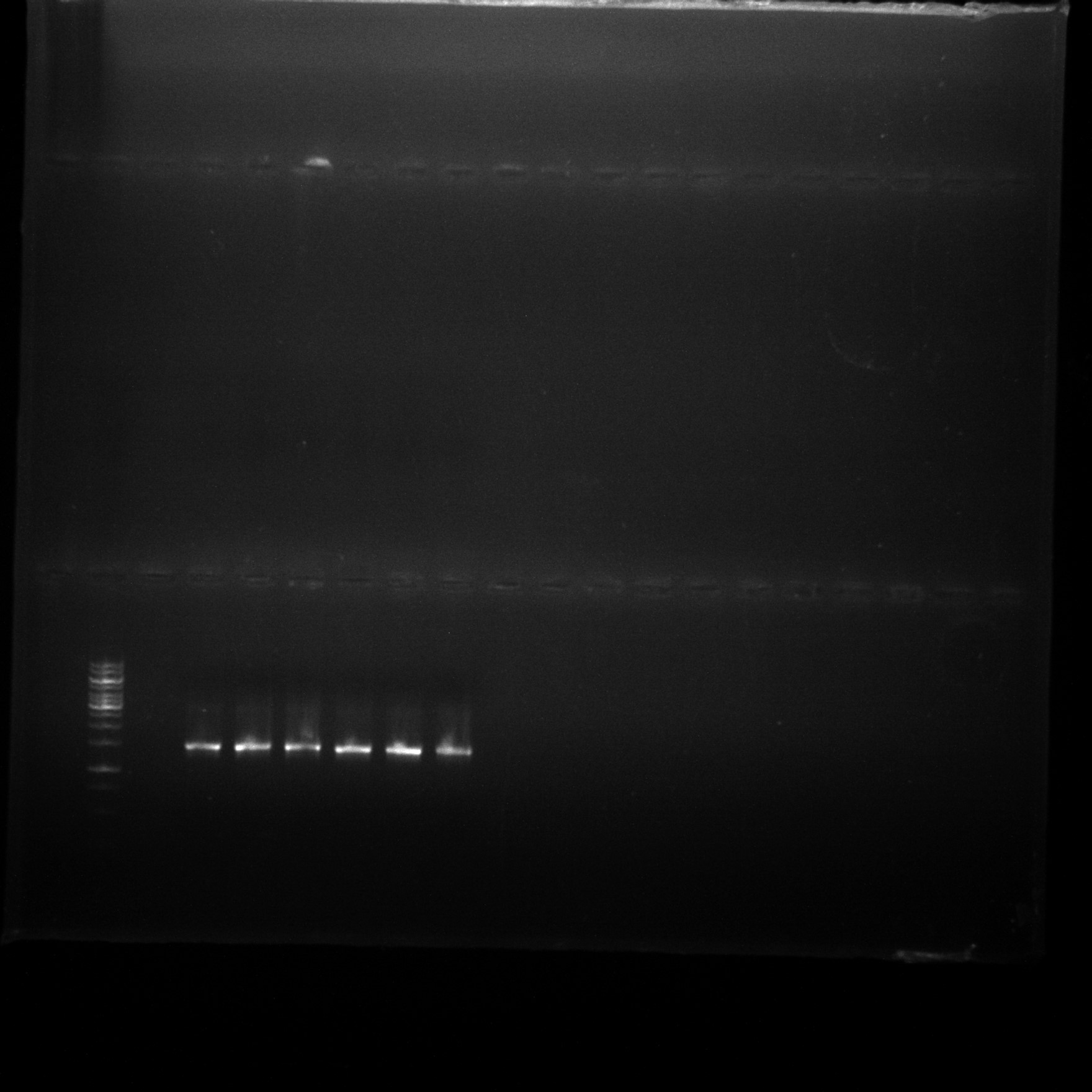

Supplement: Supplemental Information 2 [file peerj-11-14621-s002.jpg]
